# An Insight into the Stages of Ion Leakage during Red Blood Cell Storage

**DOI:** 10.3390/ijms22062885

**Published:** 2021-03-12

**Authors:** Anna Zimna, Magdalena Kaczmarska, Ewa Szczesny-Malysiak, Aleksandra Wajda, Katarzyna Bulat, Fatih Celal Alcicek, Malgorzata Zygmunt, Tomasz Sacha, Katarzyna Maria Marzec

**Affiliations:** 1 Jagiellonian Centre for Experimental Therapeutics, Jagiellonian University, 14 Bobrzynskiego St., 30-348 Krakow, Poland; a.zimna@student.uj.edu.pl (A.Z.); ewa.szczesny@jcet.eu (E.S.-M.); olawajda@agh.edu.pl (A.W.); katarzyna.bulat@jcet.eu (K.B.); f.celal.alcicek@jcet.eu (F.C.A.); 2 Faculty of Pharmacy, Jagiellonian University Medical College, 9 Medyczna St., 30-688 Krakow, Poland; malgorzata.zygmunt@uj.edu.pl; 3 Faculty of Materials Science and Ceramics, AGH University of Science and Technology, 30 Mickiewicza St., 30-059 Krakow, Poland; 4 Chair of Haematology, Faculty of Medicine, Jagiellonian University Medical College, 12 sw. Anny St., 30-008 Krakow, Poland; tomasz.sacha@uj.edu.pl; 5 Department of Haematology, Jagiellonian University Hospital, 17 Kopernika St., 31-501 Krakow, Poland

**Keywords:** red blood cell, membrane ions transport, red cell aging, atomic force microscopy

## Abstract

Packed red blood cells (pRBCs), the most commonly transfused blood product, are exposed to environmental disruptions during storage in blood banks. In this study, temporal sequence of changes in the ion exchange in pRBCs was analyzed. Standard techniques commonly used in electrolyte measurements were implemented. The relationship between ion exchange and red blood cells (RBCs) morphology was assessed with use of atomic force microscopy with reference to morphological parameters. Variations observed in the Na^+^, K^+^, Cl^−^, H^+^, HCO_3_^−^, and lactate ions concentration show a complete picture of singly-charged ion changes in pRBCs during storage. Correlation between the rate of ion changes and blood group type, regarding the limitations of our research, suggested, that group 0 is the most sensitive to the time-dependent ionic changes. Additionally, the impact of irreversible changes in ion exchange on the RBCs membrane was observed in nanoscale. Results demonstrate that the level of ion leakage that leads to destructive alterations in biochemical and morphological properties of pRBCs depend on the storage timepoint.

## 1. Introduction

The main function of the red blood cells (RBCs) is to carry respiratory gases, especially oxygen and carbon dioxide through the circulatory system. Gas exchange is possible because of the presence of haemoglobin (Hb) in the cytosol of RBCs [1]. The most common states of Hb are deoxyhaemoglobin (deoxyHb) and oxyhaemoglobin (oxyHb). To make the process of gas exchange efficient, human RBCs developed a special biconcave shape, which enlarges the surface area to volume ratio, and, therefore, diffusion is much more effective. The biconcave shape of the RBC is maintained and stabilized by its cytoskeleton, composed of spectrin, F-actin, ankyrin, band 4.1, and other proteins that connect RBC’s skeleton with its membrane through junctional complexes. Such complexes mainly comprise protein band 3, glycophorin A, adducin, and glucose transporter 1 (GLUT1) [2]. Cell membrane plays an important role in the functionality of the RBCs, ensuring proper gas exchange function. During their average 120-day lifetime, erythrocytes are exposed to various environmental changes and different sorts of stress, which can result in cell damage, i.e., disruption of cell integrity, morphology and function, structural changes, and even death (haemolysis) [3,4,5,6,7]. The main role of the RBC membrane, which is a selective barrier between the RBC interior and external environment, is to maintain the stability of the components in both compartments. The regulation of ion exchange is possible because the RBC membrane takes part not only in gas transport, but also in the regulation of pH and water content inside the RBC [3]. Proper ion exchange via cell membranes enables maintenance of RBCs biconcave disk shape and stable flexibility [8]. On the other hand, RBC adaptation to environmental changes can be modified by the number of exchanged ions. Depending on the ion type, different transporters are activated. Cations, mainly Na^+^, K^+^, Li^+^, Mg^2+^, and Ca^2+^, and even those of heavy metals like cesium (Cs), activate mechanosensitive ion channel called Piezo1 and, thus, stimulate their influx to the cell [9,10,11]. On the contrary, the Gardos channel, which is activated by calcium ions, activates the efflux of potassium [12,13,14]. Additionally, there are nonselective ion channels that also regulate the cation concentrations, e.g., Calcium-inhibited Cation Channel (CiCC). Its conduction increases in the absence of Ca^2+^, leading to disruption in K^+^ and Na^+^ concentrations, as they are transported outward and inward, respectively [15]. Another example is a nonselective voltage-activated cation channel, which is permeable for monovalent cations. It is dependent of extracellular saline, whose increased concentration promotes more pronounced flux of Na^+^ and K^+^ [16,17]. The protein responsible for the regulation of the levels of Cl^−^ and HCO_3_^−^ is called capnophorin, also known as band 3 [18]. Band 3 covers approximately 26% of the membrane surface [19] and performs chloride shift, which is crucial for gas exchange. In tissues, CO_2_ diffuses into the RBC and is rapidly hydrated to H_2_CO_3_ and dissociated to H^+^ and HCO_3_^−^ in the presence of carbonic anhydrase. Subsequently, band 3 exchanges intracellular HCO_3_^−^ for extracellular Cl^−^ (chloride shift). Intracellular acidification, occurring during the CO_2_ diffusion, triggers the dissociation of O_2_ from oxyHb and, therefore, tissues that produced CO_2_ in course of their metabolism are supplied with O_2_. Acceptance of protons by reactive groups of deoxyHb and the occurrence of the Bohr effect restores the pH and prevents O_2_ from further dissociation [20]. Furthermore, two main pumps regulated by the intracellular ATP include: Na^+^-K^+^-ATPase and Ca^2+^-ATPase. Both produce adenosine diphosphate, which is later used in glycolysis to produce energy. The process of glycolysis begins with glucose uptake by GLUT1 transporter. Later, glucose is modified in various biochemical reactions to finally produce lactates. This product of glycolysis is transported across the membrane of the RBCs through nonionic diffusion, via band 3 anion exchanger and monocarboxylate carrier systems (MCTs) [21,22,23]. Moreover, increased extracellular concentration of lactate ions (La^−^) produced during accelerated glycolysis stimulates higher influx of cations through Piezo1 [10].

A donor’s blood may contain immature RBCs’ called reticulocytes (RET) and RBCs that are up to average 120 days old [24]. Therefore, present studies were focused on the ions regulated by Na^+^-K^+^ pump (increased activity, more pump units in RET) [25]; Na^+^-K^+^ co-transport (decreased in RET, increased in mature RBCs); K^+^-Cl^−^co-transport, Na^+^/H^+^ exchanger (both increased in RET, decreased in mature RBCs) [26,27,28,29]; Na^+^-K^+^-2Cl^−^ counter transport (increased in mature cells) [30,31], and band 3, also known as the anion exchanger 1, whose level increases during cell maturation [32,33]. The latter regulates the influx of Cl^−^, efflux of HCO_3_^−^, contributes to H^+^ concentration, and is known to have a decreased activity, not only when storage lesions occur, but also during RBCs aging due to aggregation [34]. The main goal of this work was to analyze the temporal sequence of the variation in concentrations of singly charged ions, namely Na^+^, K^+^, Cl^−^, H^+^, La^−^, HCO_3_^−^ in human packed RBCs (pRBCs), to analyze the relationship between ion exchange and biochemical changes together with RBCs morphology. We have applied the combination of different physical and chemical methods of RBCs analysis, which allowed us for a detailed description of ion exchange supported by intra- and extracellular measurements of the biochemical, morphological and functional changes, and their influence on gas exchange.

## 2. Results and Discussion

### 2.1. Analysis of the Ion Leakage Stages during pRBCs Storage

**Figure 1:**
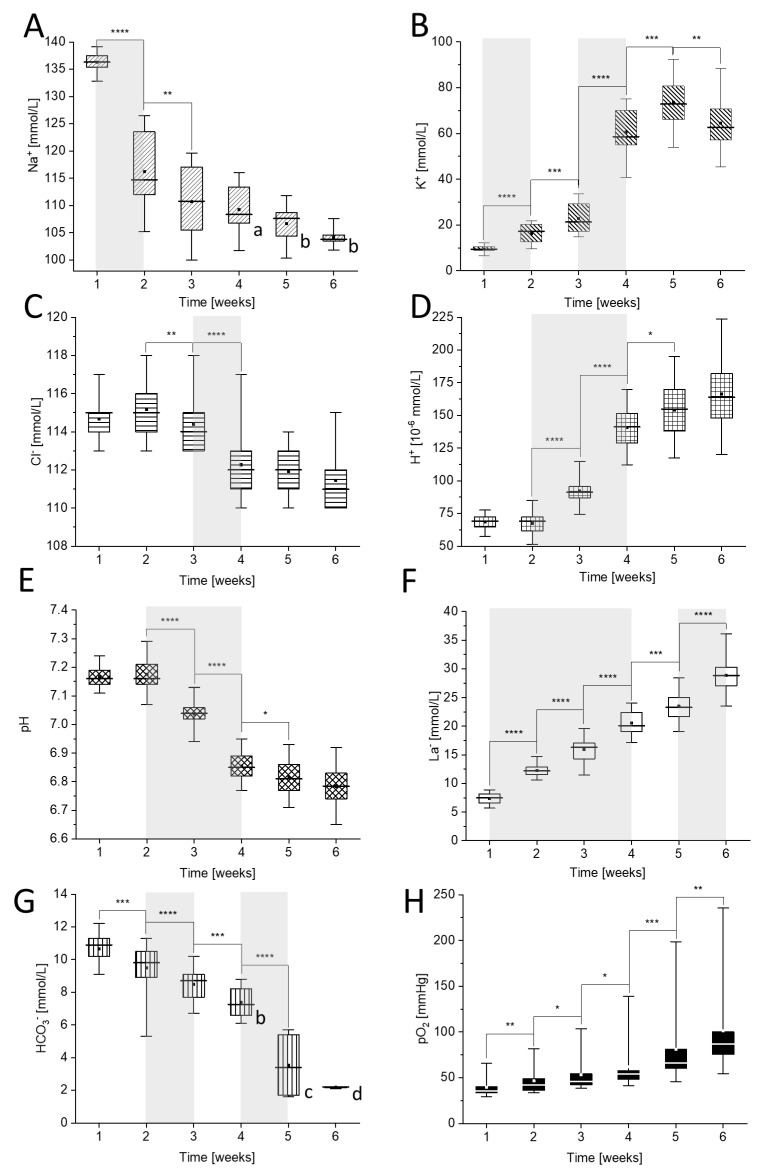
Time-dependent changes in: (**A**) [Na^+^], (**B**) [K^+^], (**C**) [Cl^−^], (**D**) [H^+^], (**E**) pH, (**F**) [La^−^], (**G**) [HCO_3_^−^], and (**H**) pO_2_ during storage of packed red blood cells (pRBCs). Measurements were carried out weekly for six weeks. Letters a–d mark the results obtained for the following number of donors: (a) *n* = 8, (b) *n* = 7, (c) *n* = 4, (d) *n* = 2, while other were obtained from eleven donors (*n* = 11). The most prominent changes are marked gray. Data distribution is presented as box plots: median, Q1, Q3, interquartile range and min-max whiskers (Q1, Q3 indicate 25th and 75th percentiles, respectively). Data normality distribution was assessed using Shapiro-Wilk test. Statistical significance of the obtained values was tested with Kruskal-Wallis ANOVA nonparametric test (null = not significant; * *p* < 0.05; ** *p* < 0.01, *** *p* < 0.001, **** *p* < 0.0001).

As shown in Figure 1, a gradual increase in [K^+^], [H^+^], [La^−^], and pO_2_, together with a decrease in [Na^+^], [Cl^−^], [HCO_3_^−^], and pH were observed during six weeks of pRBCs storage. Between the first and the second week of storage, a 15% drop in sodium values was noticed (*p* < 10^−4^, Figure 1A) and it corresponded with the decrease in Na^+^ concentration from ~135 mmol/L to ~115 mmol/L. Simultaneously, the concentration of K^+^ (*p* < 10^−4^, Figure 1B) increased from ~10 mmol/L to ~16 mmol/L in contrast to [HCO_3_^−^] (*p* < 10^−3^, Figure 1G) that decreased from ~10.5 mmol/L to ~9.3 mmol/L, which was accompanied with the statistically significant increase of [La^−^] (*p* < 10^−4^, Figure 1F) from ~7.5 mmol/L to ~12.5 mmol/L and increase in pO_2_ (*p* < 10^−2^, Figure 1H) from ~39 mmHg to ~46 mmHg. We did not observe any statistically significant changes in case of [Cl^−^] and [H^+^], nor in pH during the first week of storage.

Between the second and the third week of storage, the trends observed in the first week for [K^+^], [Na^+^], and [HCO_3_^−^] as well as for [La^−^] and pO_2_ persist. Additionally, statistically significant decrease in [Cl^−^] (*p* < 10^−2^, Figure 1C) and a substantial increase in [H^+^] from around 68 × 10^−6^ mmol/L to around 92 × 10^−6^ mmol/L (*p* < 10^−4^, Figure 1D) with a corresponding decrease in pH from around 7.2 to around 7.05 (*p* < 10^−4^, Figure 1E) were observed. Between the third and the fourth week of storage, a decrease in [Na^+^] slowed down. At the same time, the largest increase in [K^+^] and [La^−^] was observed, from ~23 mmol/L to ~61 mmol/L (*p* < 10^−4^, Figure 1B) and from ~15 mmol/L to ~21 mmol/L (*p* < 10^−4^, Figure 1F), respectively. Moreover, the largest decrease in [Cl^−^] (*p* < 10^−4^, Figure 1C) from ~114.5 mmol/L to ~112.3 mmol/L occurred. Prominent changes were observed also in [HCO_3_^−^] and [H^+^], pH and pO_2_. Between weeks 4 and 5, the largest decrease occurred in [HCO_3_^−^], from ~7.3 mmol/L to ~3.6 mmol/L (*p* < 10^−4^, Figure 1G). It was accompanied by a significant increase in pO_2_ from ~61 mmHg to ~81 mmHg (*p* < 10^−3^, Figure 1H) and an increase in [La^−^] from ~20 mmol/L to ~22 mmol/L (*p* < 10^−3^, Figure 1F). An increase in [K^+^] and [H^+^] was still significant, while changes in other ions’ concentrations slowed down. At the end of storage, between the fifth and the sixth week, we did not observe statistically significant changes in ion concentrations, except for a decrease in [K^+^] accompanied by an increase in pO_2_ (*p* < 10^−2^, Figure 1H) and continuation of the significant increase in [La^−^] leakage (*p* < 10^−4^, Figure 1F).

We may conclude that these results show a complete picture of RBC ion changes and pRBC environment disturbance, beginning with a vast decrease in [Na^+^] after the first week of storage, followed by a decrease in [Cl] after the second week, an increase in [H^+^] accompanied by a pH decrease between the second and the fourth week, an increase in [K^+^] after the third week and a decrease in [HCO_3_^−^] after the fourth week. Additionally, a constant increase (statistically significant for each week of storage) in [La^−^] and pO_2_ was observed during the whole time of storage. Furthermore, the comparison between results presented in Figure 1 and the reference values of ions’ concentration observed in human blood in vivo [35,36,37] (collected in Tables S2 and S3) clearly prove that most ions studied in pRBCs fell out of the reference range. At the end of the experiment, concentrations of Na^+^, H^+^, and HCO_3_^−^ were below the reference range. The same applies to [K^+^]. Only the variation of [Cl^−^] in pRBCs stayed within the range of the in vivo reference values. 

As had previously been reported [38,39] and confirmed in this work, lactate level significantly increases with the time of storage (Figure 1F), while the glucose level decreases (Table S3). Such changes in glucose and lactate (resulting from glycolysis) systematically continue to occur in pRBCs during the whole time of storage. However, as has previously been reported, the storage of pRBCs at 4 °C slows down the production of ATP, what is also related to ion exchanger disturbance [24,40]. According to the Post-Albers scheme, with normal metabolism, Na^+^-K^+^ pump exchanges 3 intracellular Na^+^ for 2 extracellular K^+^ [41]. In our research, a rapid 15% decrease in [Na^+^], accompanied by a significant increase in [K^+^] are observed during the first and the second week of pRBCs storage, which suggests the disturbance of ion exchange. As previously reported, the RET are characterized by higher K^+^ turnover and higher Na^+^ leakage, while older RBCs show increased K^+^ efflux [28,30,31,42]. These data are in accordance with our study, as the fraction of RET is the highest in pRBCs at the beginning of storage, which is reflected by the lowest [Na^+^] efflux in first and the second week of pRBCs storage. On the other hand, an acceleration of increase in [K^+^] leakage in the third week may be related to an increased number of mature RBCs.

A drop in pH values is caused by more intense lactic acid synthesis, starting from the first week of cold storage, which is also a reason why glycolysis slows down, since it is inhibited via a negative feedback mechanism [43]. During the significant change of pH between the second and the third week (Figure 1E), a slowdown of subsequent ionic transporters is also observed. Along with pH decrease from 7.15 to 6.8, the water content of RBCs is affected [44], which indirectly influences the passive transport through the membrane and increases cell hydration [24]. In consequence, between the third and the fourth week, [K^+^] increase remarkably, which is accompanied by a simultaneous drop in the [Cl^−^] (Figure 1B,C). This points to the disruption, not only of the K^+^/Cl^−^ cotransporters [25,45], but also other nonselective cation channels such as CiCC or voltage-activated cation channel. We may state that low level of extracellular [Ca^2+^] is related to chelation of Ca^2+^ ions with citrate present in additive solution containing citrate, phosphate, dextrose-saline, adenine, glucose, mannitol (CPD–SAGM). This, in turn, may contribute to a drop in [Na^+^] and an increase in [K^+^] seen during storage, as CiCC has been linked to higher extracellular [K^+^] and lower extracellular [Na^+^], which was previously described as a cause of storage lesion, i.e., morphological changes and vesiculation [15,46]. As transfusion bags are buffered with CPD–SAGM, they have high saline concentration. Higher extracellular NaCl concentration promotes opening of a nonselective cation channel, which influences flux of the monovalent cations, mainly Na^+^ inward and K^+^ outwards. Once activated, the channel stays open even after normalization of saline concentration. The same mechanism can be triggered by membrane deformations—it also induces nonselective cation channel opening [16,17].

pH also influences oxygen carrying affinity of Hb by lowering the concentration of 2,3-bisphosphoglycerate (2,3-BPG), and, therefore, it disrupts functional properties of the pRBCs’, namely gas exchange [47], which was shown in our additional studies revealing correlations between changes in deoxyHb and oxyHb levels in weeks 3 and 4 (Figure S1).

It was previously shown that due to band 3 action, when pO_2_ pressure increases, the amount of the HCO_3_^−^ anions decreases [48] and the same trend is observed in our research of pRBCs storage. The process of band 3-dependent Cl^−^/HCO_3_^−^ influx seems to begin during the second week of storage, when a significant decrease in pH and [HCO_3_^−^] occurs (Figure 1E,G), and is accompanied by an increase in pO_2_ (Figure 1H). Furthermore, throughout the third and the fourth week, a drop in [Cl^−^] was observed. All this data stands in line with previous studies, where the influence of SAGM solution on pRBCs was measured. It was concluded that SAGM solution induces a faster decrease in [Cl^−^] and pH, and, therefore, promotes chloride shift via band 3 [47]. A pronounced increase in pO_2_ (Figure 1H) leads to statistically significant drop in [HCO_3_^−^] (Figure 1G). Respectively, a downfall in chloride concentration (Figure 1C) ceases in the fifth week, when a noticeable reduction in [HCO_3_^−^] can be observed (Figure 1G). Furthermore, a rather constant [Cl^−^] can be a result of band 3 depletion. As we have presented further on in Chapter 2.3. with the application of atomic force microscopy (AFM) and results published in our previous work [38], the disturbance of band 3 is also confirmed by formation of echinocytic morphology and increased microvesicle release, providing another mechanism for vesiculation [49]. Hence, a steady concentration of Cl^−^ and HCO_3_^−^ after the fifth week can be an effect of a decrease in both the amount and activity of band 3 in the membrane.

### 2.2. The Iimpact of Blood Group Type on the Ion Concentration

**Figure 2:**
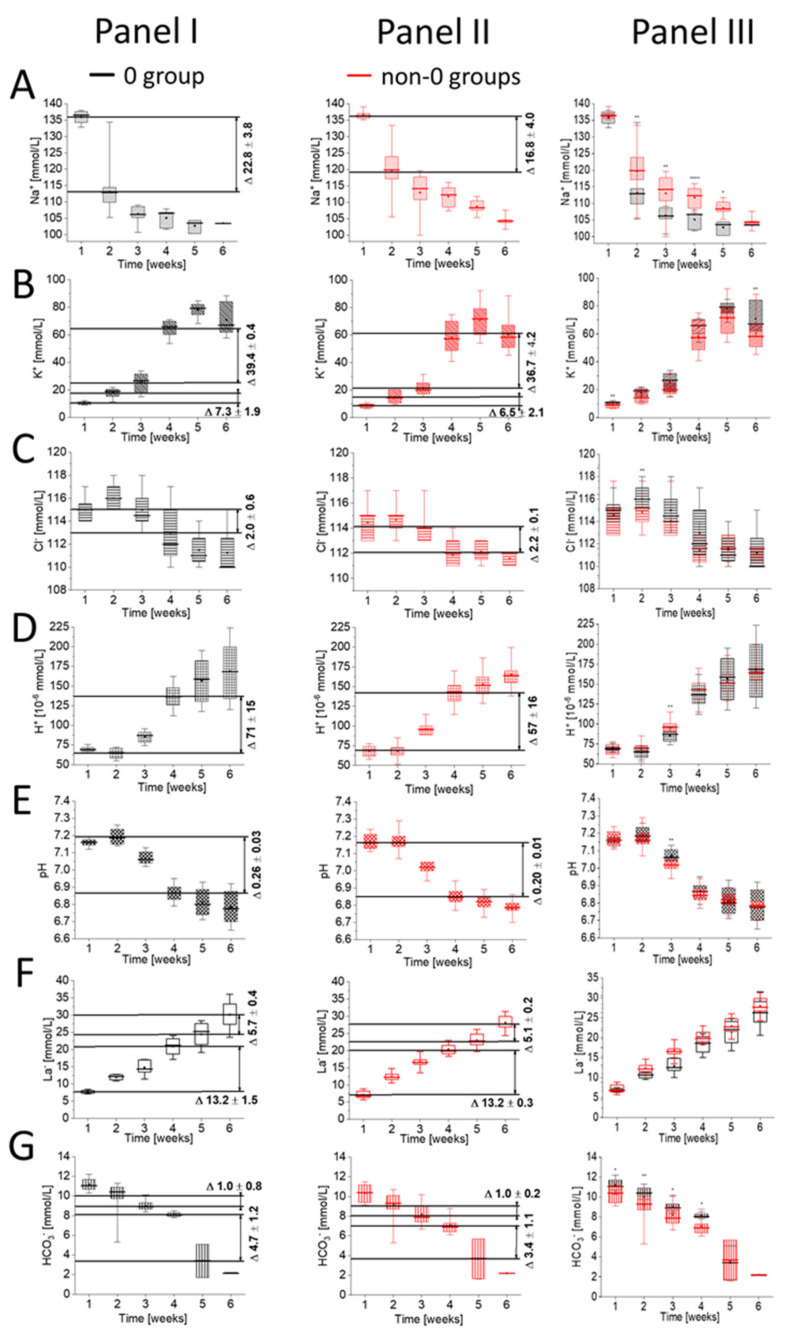
Comparison of time-dependent changes in (**A**) Na^+^, (**B**) K^+^, (**C**) Cl^-^, (**D**) H^+^, (**E**) pH, (**F**) La^−^ and (G) HCO_3_^−^ concentrations between 0 (Panel I) and non-0 (Panel II) blood groups with the difference of values (∆) marked for each measured parameter in ranges marked in gray in Figure 1, which correspond only to substantial concentration changes (*p* < 10^−4^). Results are given for *n* = 4 (group 0) and *n* = 7 (group non-0). The non-0 group contains pRBCs obtained from the donors with A group (*n* = 4) and with B group (*n* = 1) and AB group (*n* = 2). Data distribution is presented as box plots: median, Q1, Q3, interquartile range, and min-max whiskers (Q1, Q3 indicate 25th and 75th percentiles, respectively). Data normality distribution was assessed using Shapiro-Wilk test. Panel III—Statistical significance of the obtained values for 0 and non-0 groups in each week was tested with KruskalWallis ANOVA nonparametric test (null = not significant; * *p* < 0.05; ** *p* < 0.01, *** *p* < 0.001, **** *p* < 0.0001).

Figure 2 presents the temporal sequence of changes in the ion exchange in pRBCs, according to the blood group type of studied samples. The results are given for group 0 (*n* = 4) and non-0 group (n = 7). The non-0 group contains pRBCs obtained from the donors with A group (*n* = 4), B group (*n* = 1), and with AB group (*n* = 2). The lack of bigger sample size of particular blood groups for comparison presented in Figure 2 is a clear limitation of these results.

Every ABO antigen present on the surface of the RBCs’ membrane originates from a different type of carbohydrates and defines a specific blood type. Group 0 consists of fucose on membrane glycoprotein, which determines antigen H. Group A additionally contains N-acetylgalactosamine, which determines antigen A. Group AB consists of antigen A plus antigen B, which is made of galactose on the H chain [50]. The role of these antigens is obvious in serology and transfusion; however, there are researchers concerned with a link between susceptibility to some diseases, such as malaria or hereditary spherocytosis, and certain blood groups, e.g., the ABO antigens with transmembrane proteins that comprise transporters [51,52,53,54,55].

Changes in ions presented in Figure 1 are still noticeable for each blood type; however, the rate of ion changes and the time of the most prominent changes vary between blood groups. The result comparison presented in Figure 2 reveals the greatest sensitivity of group 0 to time-dependent ionic changes when compared to other non-0 blood groups.

The comparison of substantial concentration changes (*p* <10^−4^) presented as ∆ values in Figure 2 Panel I, Panel II reveals that these values are the highest in the case of blood type 0. For Na^+^, K^+^ and HCO_3_^-^ concentrations, ∆ values showed statistical reduction in case of group 0 in comparison to a decrease in non-0 groups (Figure 2A,B,G Panel I and Panel II). In these cases, changes observed between group 0 and non-0 groups were statistically relevant in almost every week of the storage (Figure 2A,B,G Panel III). Figure 2C–E Panel III reveal that significant differences were observed in the variation of [K^+^], [Cl^−^] and [H^+^] (pH) only during a single week of the storage, different for each ion. In case of [La^−^] (Figure 2F Panel III), no significance was noticed.

Cold, long-term pRBC storage causes higher ion permeability of the RBC membrane, with significant lactate accumulation. In our study, changes in [Na^+^], [K^+^] and [HCO_3_^−^] were most evident in group 0 (Figure 2A,B,G), and could indicate greater resistance of group 0 to the changes occurring during pRBC storage. One of the causes of such behavior can be related to changes in the cationic environment of RBCs of 0 type. The fastest changes in ion levels due to their leakage, especially [Na^+^] and [K^+^], were demonstrated for group 0. It seems that RBCs of group 0 respond faster to the reduction of the ion transporters’ activity (e.g. Na^+^-K^+^ pump), which in result promotes highest Na^+^ influx to the RBC, while K^+^ significantly increases in the extracellular milieu, in comparison to the RBCs of non-0 groups. This can be an indication of adaptation to conditions where group 0 gained the ability to perform quicker changes in ionic concentrations, and as a result, adapt more easily to environmental changes. Additionally, HCO_3_^−^ concentration dropped remarkably in group 0 (Figure 2G). This can also indicate abovementioned adaptation of band 3 anion exchanger, which is revealed in our study as a faster and more rapid change in [HCO_3_^−^] due to storage, and as a presence of membrane alterations. However, such conclusion related to group-0 should be considered in the relation to the presented limitations of our research, and only further research comprising a larger group of donors including female donors can confirm such findings.

### 2.3. Relationship between Ion Exchange and RBCs Morphology

We have previously reported that ion exchange also influences the shape and functionality of pRBCs [38]. In anionic or non-ionized solutions, the RBCs become crenated (and known by the names of echinocytes), while in cationic solutions they become cup-like (stomatocytes) [56]. As it was already reported, with a decrease in pH (and addition of hypotonic media) more cup-like cells such as stomatocytes [56] are formed.

**Figure 3:**
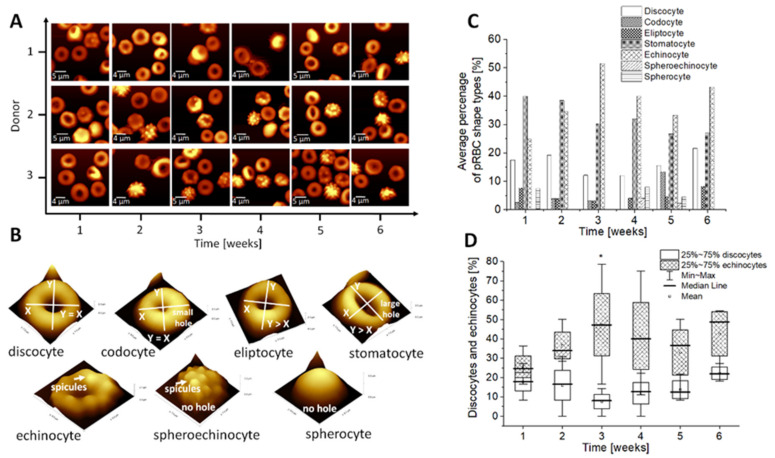
(**A**) Representative 2D AFM images obtained during analysis of dry smears; (**B**) Example of 3D AFM images of different red blood cell (RBC) shape types observed during storage; (**C**) Time-dependent changes of the discocytes and stomatocytes observed weekly for six weeks of storage in pRBCs. Data distribution is presented as box plots (median, Q1, Q3, interquartile range, min-max whiskers). Q1, Q3 indicate 25th and 75th percentiles, respectively. Statistical significance of the obtained data (*n* = 3) was tested with Kruskal-Wallis ANOVA nonparametric test followed by Tukey’s post-hoc (*p < 0.05); (**D**) Analysis of RBC shape types as presented in (**B**) based on nanoscale AFM measurements analyzed weekly during six weeks of storage of pRBCs (*n* = 3). Besides statistically significant difference between discocytes and stomatocytes presented in (**C**) for the second week of storage, other results in (**D**) were found not significant.

Figure 3 presents the AFM analysis of dry smears of pRBCs (Figure 3A), which allowed for the nanoscale analysis of the chosen types of pRBCs shapes (Figure 3B) during their transformation caused by changes in the ionic environment. In our studies, we observed that throughout the whole pRBC storage period, discoidal RBCs took different forms: starting from stomatocytes characterized by an oval or rectangular central pallor, through spiculated echinocytes, ending with spheroechinocytes and spherocytes with dysfunctional spherical shape. The analysis of the average percentage of the pRBC shape types observed weekly for studied dry smears suggested donor (*n* = 3) dependent variation of different shape types (Figure 3C). The statistically significant results were obtained only for the visible increase of echinocytes in third week of storage in comparison to discocytes as revealed by analysis presented in Figure 3D. As revealed in Figure 3B, the greatest increase in the number of echinocytes and a simultaneous decrease in stomatocytes was observed between the second and the fourth week of storage, when the pH showed a significant drop (Figure 1E). Changes observed in RBCs morphology seem to be a result of changes in intracellular and extracellular pH values, which depend on anion transfer. Another theory is that the biconcave shape is sustained when the pH gradient between membrane and medium is below a constant, critical value [56]. As mentioned before, the shape of erythrocytes is modified by changes in the ionic environment. Ions work in different ways, promoting different pathways of deformation of the discoid RBCs. They can either be adsorbed on the outer layer of the membrane or penetrate inside the cell and induce changes in the tension and conformation of lipoproteins [9]. Anions will bind to cationic groups, while cations will react with anionic groups. Moreover, some cations can interfere with calcium binding in the membranes, i.e. during stomatocyte formation [56]. Moreover, band 3 is not only a transporter for Cl^−^/HCO_3_^−^ but also a protein important for the maintenance of the red cell structure and morphology. It links the membrane to the underlying spectrin cytoskeleton via a strong bonding with ankyrin. The segment anchored to the cytoskeleton does not change during aging. The honeycomb shape of the membrane skeleton seems to be preserved (Figure S2). However, due to oxidative stress and peroxidation, the RBC membrane gets disrupted, which leads to a loss of phospholipids via vesiculation, along with immobilization of band 3 segments [57]. The presence of microvesicles on the RBCs’ surface was observed in our AFM studies (Figure S2). It is suggested that phosphorylation of band 3 disrupts its interaction with ankyrin and this way weakens the membrane-cytoskeleton association [49], leading to modifications of cell morphology and the occurrence of reversible or irreversible effects on the cell and membrane properties [58,59]. The high state of band 3 phosphorylation was shown to result in echinocytic morphology and increased microvesicle release, providing another mechanism for vesiculation [49], leading to a decrease in the cholesterol and triglycerides levels in RBCs membrane and in consequence destabilizing their membrane structure, which we have observed in our previous studies [38]. AFM results together with the ion leakage presented in chapter 2.1. suggest the disruption of band 3 and RBC hydration alterations.

### 2.4. Relationship between Ion Exchange and Red Cell Quality Indices

To link alterations in ion leakage with RBC morphological changes, the RBCs quality indices (mean corpuscular volume (MCV), RBC, and haemoglobin concentration (HGB)) were analyzed. Additionally, to show progression of the haemolysis of the stored RBCs or/and Hb leakage, in each week the fFe was assessed in supernatant.

**Figure 4:**
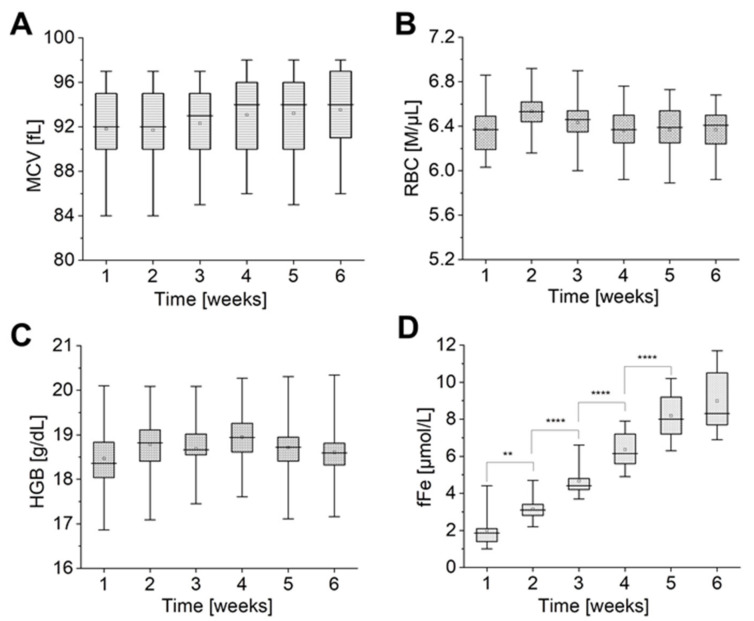
Time-dependent changes in red cell quality indices: (**A**) mean corpuscular volume (MCV), (**B**) RBC and (**C**) haemoglobin concentration (HGB) and (**D**) fFe during pRBCs storage (*n* = 11). Data distribution is presented as box plots: median, Q1, Q3, interquartile range, and min-max whiskers (Q1, Q3 indicate 25th and 75th percentiles, respectively). Data normality distribution was assessed using Shapiro-Wilk test. Statistical significance of the obtained values was tested with Kruskal-Wallis ANOVA nonparametric test (null = not significant; ** *p* < 0.01, **** *p* < 0.0001).

As shown in Figure 4A–C, the mean values of MCV, RBC, and HGB parameters fluctuate at the levels of ~92–94 fL, ~6.4–6.5 M/μL, ~18.5–19 g/dL, respectively, while values of fFe constantly increase (Figure 4D) during the whole period of storage.

No statistically significant change was observed in MCV, RBC, and HGB (Figure 4A–C). Average MCV, RBC, and HGB values remained at the same level until the last week of the storage, pointing to a lack of changes in dependent parameters, such as: mean corpuscular haemoglobin (MCH = HGB/RBC) and mean corpuscular haemoglobin concentration (MCHC = (HGB × 1000)/(MCV × RBC)). However, it is important to state that MCV values showed a large individual variation in the change kinetics, with some donors showing a significant increase in MCV, when some showed no change during the time of storage, which altogether is observed as a slight tendency of increase the MCV mean value. Those observations were accompanied by a statistically significant rise of fFe values, which was persistent until the fifth week of pRBCs storage. In the sixth week, no significant changes were noticed in free iron concentration.

On the one hand, it was previously reported that there is no significant change to be observed in MCV, HGB, and RBC, during the fourth week of storage [60,61]. On the other hand, there are many studies reporting an increase in MCV accompanied by a decrease in MCHC values and an increase in RBCs haemolysis during six weeks of storage [62,63,64,65]. It results from a deregulated mechanism of cell volume maintenance and points to the distinct correlation of the altered cation gradient across the RBC membrane with the changes of cell volume and shape during pRBC storage [24,66].

Our measurements share some similarities with observations from previous studies. An upward trend observed for MCV value (Figure 4A) could be correlated with a simultaneous decrease in [Na^+^] and an increase in [K^+^] (Figure 1A,B) and, as previously reported, with an increase in water influx to RBCs [62]. Those alterations also revealed themselves as shape changes of RBCs, as shown by AFM results (Figure 3B): from discoidal to spiculated and spherical forms of the RBCs. This may relate the slight increase in MCV to the increase in [Na^+^] in RBC, which may cause swelling of cells due to “water follows sodium” physiological rule [37], leading to a decrease in [K^+^] in the RBC. No significant changes were observed for the HGB and RBC values during pRBCs’ storage (Figure 4B,C), which correlates with previous studies [62]. The results point to stability of the MCH indicator and suggest no significant effects on the RBC integrity. A significant increase in free iron (Figure 4D) indicates the progression of the vesiculation process and points to progressing haemolysis and Hb leakage to supernatant. The loss of the RBC membrane through vesiculation makes the stored RBCs become smaller and spherocytic. In this situation, to maintain in an increase in the MCV, the remaining RBC must be even more swollen, which correspond with previous studies [24,44,64,66] and our AFM results (Figure 3).

*Research Limitation:* Our studies reported, herein, should be considered in the light of some limitations. Our experimental setup was based on eleven healthy male donors, with BMI in the range of 18.5 to 35.0, in the prime of life, i.e., between 26 and 60 years and an average of 38 years of age. The results are given for group 0 (*n* = 4; ages 26,30,32,38) and non-0 group (*n* = 7). The non-0 group contains pRBCs obtained from donors with A group (*n* = 4; ages 28,35,39,43), B group (*n* = 1; age 44) and AB group (*n* = 2; ages 40,60). The number of donors clearly has an impact on the findings presented and analyzed in Figure 2. The conclusions should be drawn in relation to the relatively small number of donors, who are, however, of comparable age and BMI. Moreover, the pRBCs were purchased commercially and we had limited access to information about donors other than age, sex, blood group, and hemoglobin concentration. Major limitations of this study could be addressed in future research. To confirm our findings, additional research needs to be performed with an increased number of donors, including females.

## 3. Materials and Methods

### 3.1. Sample Preparation

Leukodepleted pRBCs withdrawn from healthy male donors were purchased from Regional Centre for Blood Donation and Haemotherapy in Krakow (*n* = 11). Informed consent was given by each volunteer prior to the blood withdrawal and the study conformed with the principles outlined in the World Medical Association (WMA) Declaration of Helsinki, as well as Bioethical Commission of Jagiellonian University.

pRBCs were suspended in additive solution (CPD–SAGM). Measurements of RBCs aging were carried out for each bag every seven days, for six weeks. During that time, pRBCs were stored at 4–6 °C in accordance with WHO recommendations.

### 3.2. Ions and Blood Gases Measurements

The measurement of potassium ions was conducted with the use of an ion-selective electrode (DETECTOR s.c., Raszyn, Poland) consisting of a double-junction reference electrode (with reference solutions 1.0 mol/L KCl and 0.1 mol/L (NH_4_)_2_SO_4_) and a detector set on the proper settings (the measurement in pX, mmol/L) both connected to a multimeter type CX-601 (ELMETRON, Zabrze, Poland). Every week at the beginning of the measurements, the ion-selective electrode was filled with reference solutions: 1 mol/L KCl and 0.1 mol/L (NH_4_)_2_SO_4_ and conditioned in a standard solution of 0.1 M KCl for 15 min. Then, calibration was conducted as measurements of four standard KCl solutions: 1 mM, 10 mM, 100 mM, and 1 M. The measurement of each sample was performed two times after electrode stabilization.

The pH of each sample was measured two times using electrode type ERH-11S (HYDROMET s.c., Gliwice, Poland) and a multimeter type CX-601(ELMETRON, Zabrze, Poland). The pH meter was fitted with a temperature probe. Before each series of measurements, 3-point electrode calibration was conducted using 3 different buffer solutions. Additionally, the pH values were recalculated on the base of hydrogen H^+^ ions concentration, based on the following equation: [H^+^] = 10^−pH^ × 1000 [mmol/L].

The concentration of sodium Na^+^, chloride Cl^−^, bicarbonate HCO_3_^−^ ions were measured with the use of SIEMENS RAPIDPoint 500 Analyzer (Siemens Healthcare, Sudbury, UK). The same device was used for measurements of partial pressure of O_2_ (pO_2_) and oxyHb and deoxyHb percentage. The samples were tested three times each, immediately after being taken from the pRBC bag.

Biochemical tests of La^−^ and free iron (fFe) concentrations were conducted in the additive solution obtained by two centrifugations of pRBCs samples at room temperature: the first one on 500× *g* for 10 min and the second on 3000× *g* for 5 min. The ABX Penta 400 (Horiba Medical, Kyoto, Japan) colorimetric-based biochemical analyzer and dedicated Horiba reagents were used. Necessary controls and calibrations were performed before measurements. Each sample was analyzed two times.

### 3.3. AFM Imaging

The AFM microscope (WITec, Ulm, Germany), with the highest available resolution of images of 512 × 512 lines, was used for the studies. The AFM measurements were performed on dried smears of pRBCs, at room temperature with minimized lighting (both natural and artificial). Dry smears of each pRBCs sample were studied weekly during the period of six weeks. Three AFM images of the size 25 × 25 μm^2^ were collected from random places of each smear. Just before the measurements the smears were produced with use of cells fixed for 10 min with 1% glutaraldehyde in 0.9% NaCl solution (Hct = 1%) and stored for 24 h in 4 °C as we have previously reported [67]. Fixation was performed in a way to avoid formation of advanced Heinz body-like aggregate [68]. AFM imaging of one dried smear lasted ca. 3 h. All AFM images of the RBCs were obtained in the non-contact mode (AC) using standard force modulation probes with a nominal spring constant of 2.8 N/m (WITec, Ulm, Germany). Samples were illuminated through a dry Zeiss objective type EC EPIPLAN 20×/0.4 (Carl ZeissMicroimaging GmbH, Gottingen, Germany). AFM images of 256 × 256 lines and 512 × 512 lines were collected in air conditions, from three different areas of 625 µm^2^, around ~ 64 µm^2^ and 2,25 µm^2^.

The shape of pRBCs was defined on the base of obtained AFM images of cells and literature [66,69,70]. We have differentiated the discocytes, codocytes, eliptocytes, stomatocytes, echinocytes, spheroechinocytes, and spherocytes. For each group, the specific dimensional criteria on the base of AFM results were additionally defined, comprising the diameter, height, and a height/diameter ratio (Table S1).

### 3.4. Haematologic Analysis

Morphological parameters including RBCs count (RBC), haemoglobin concentration (HGB), and mean corpuscular volume (MCV) were determined with the use of haematology analyzer (ABC Vet, Horiba, Montpellier, France). Before each measurement, pRBC samples were mixed. The samples were measured three times according to the manufacturer’s instructions.

## 4. Conclusions

In the presented work, we have shown that changes in ion levels may be linked to alterations in biochemical and morphological properties of pRBCs, progressing with storage time. Accumulation of metabolic waste, increasing with every passing week of storage causes cellular distress, seen as vast disturbances in the ion equilibrium already between the first and second week. Most of the ion concentrations measured in pRBCs fell out of the reference range for values found in the human plasma in vivo, already in the second week of storage. During pRBCs storage, a decrease in glucose level was accompanied by a vast increase in lactate, and an increase in fFe, pointing to progressing haemolysis and Hb leakage to supernatant. A rapid drop in the pH value, observed between the second and fourth week of storage, is both a result of waste accumulation, and a cause of further alterations, in Hb affinity or membrane function. The disturbances in band 3 function, were reflected in a steady concentration of Cl^−^and HCO_3_^−^ after the fifth week of storage, as well as disturbances in the shape of pRBCs observed throughout the storage period and revealed by AFM. The abovementioned phenomena lead to further changes in ion distribution, which gives an impression of a self-perpetuating vicious circle. As we have shown, a progressive loss of biochemical and morphological properties of pRBCs is mostly due to irreversible changes in ion exchange via the RBCs membrane, which in consequence leads to storage lesions. The problem of RBC storage lesions has been widely discussed in the literature, since post-transfusion complications, including multi-organ failure, are a serious threat to patients. Our results point to the need to abandon the widespread hospital practice of administering the oldest blood products, closest to their expiration date. Moreover, our work suggested that the erythrocytes of blood type 0 on the one hand adapt more easily to environmental changes, while at the same time they are more prone to undergo adverse alterations that handicap their performance as oxygen carriers. Such results should be considered in relation to the presented limitations of our research and need more studies to be confirmed. Further research comprising a larger group of donors would allow to address the problem and answer the question, whether it is necessary to establish the norms for ion concentrations in storage solutions and introduce routine testing, especially in case of type 0 donors.

## Data Availability

The data presented in this study are openly available in Jagiellonian University Repository at DOI: 10.26106/bwv5-gb97.

## Supplementary Materials

The following are available online at https://www.mdpi.com/1422-0067/22/6/2885/s1, Table S1: Table with cell naming criteria used for analysis of RBCs morphological changes during 6 weeks storage; Table S2. Reference ranges of extracellular and intracellular concentrations of Na^+^, Cl^−^ K^+^, HCO_3_^−^ and H^+^ ions in human blood in adults.; Table S3. Reference ranges of main biochemical and morphological parameters in adults.; Figure S1. Percentage changes of the oxyHb (A) and deoxyHb (B) during storage pRBCs; Figure S2. Representative nanoscale AFM images of RBCs’ surface changes collected in scale 1.5 × 1.5 μm^2^ during 6 weeks storage of the pRBCs for three donors.

## Abbreviations:

ATP	adenosine triphosphate
AFM	atomic force microscopy
2,3-BPG	2,3-bisphosphoglycerate
Ca^2+^	calcium ion
Cl^−^	chloride ion
deoxyHb	deoxyhaemoglobin
CO_2_	carbon dioxide
fFe	free iron
GLUT	glucose transporter
H^+^	hydrogen ion
Hb	haemoglobin
HCO_3_^−^	bicarbonate ion
HCT	haematocrit
HGB	haemoglobin concentration
K^+^	potassium ion
La^−^	lactate ion
Li^+^	lithium ion
MCV	mean corpuscular volume
Mg^2+^	magnesium ion
Na^+^	sodium ion
O_2_	dioxygen
oxyHb	oxyhaemoglobin
pO_2_	partial pressure of O_2_
RET	reticulocyte
RBC	red blood cell
CPD-SAGM	additive solution of citrate, phosphate, dextrose-saline, adenine, glucose, mannitol
