# Supplementary material for: An Insight into the Stages of Ion Leakage during Red Blood Cell Storage
Source: Int J Mol Sci. 2021 Mar 12;22(6):2885. doi: 10.3390/ijms22062885 (PMC7998123; doi:10.3390/ijms22062885)
Supplement: Supplementary file 1 [file ijms-22-02885-s001.pdf]

Title: An insight into the stages of ion leakage during red blood cell storage

Anna Zimna, Magdalena Kaczmarska, Ewa Szczesny-Malysiak, Aleksandra Wajda, Katarzyna Bulat, Fatih Celal Alcicek, Malgorzata Zygmunt, Tomasz Sacha, Katarzyna M. Marzec

Cell Name	Diameter 1 - Diameter 2 [µm]	Height 1 - Height 2 [µm]	Height / Diameter	Spicules
Discocyte	**≤ 0.2**	**≤ 0.3**		**no**
Eliptocyte	**≥ 1.0**			**no**
Codocyte			**> 0.35**	**no**
Stomatocyte	**< 1.0**			**no**
Echinocyte				**yes**
Spheroechinocyte			**> 0.35**	**yes**
Spherocyte			**> 0.35**	**no**

Table S1. Table with cell naming criteria used for analysis of RBCs morphological changes during 6 week storage.

Ions	Extracellular [mmol/L]	Intracellular [mmol/L]
[Na^+]	135-145	10
[Cl^-]	95-105	3
[K^+]	3.8-5.5	159
[HCO_3^-]	22-28	7
[H^+]	36×10^{-6} – 44×10^{-6}	63×10^{-6}

Table S2. Reference ranges of extracellular and intracellular concentrations of Na^+, Cl^-, K^+, HCO_3^- and H^+ ions in human blood in adults. Zapala B. et al. Laboratory Diagnostics with Elements of Clinical Biochemistry. Book, 2017:874-886; Casey JR, et al. Nat Rev Mol Cell Biol. 2010;11(1):50-61; Rhoades R. et al. Medical Physiology : Principles for Clinical Medicine. Book 2009.

Parameter	Unit	Reference range
Lactates	mmol/L	0.6-2.2
Glucose*	mmol/L	3.9-5.5
free Fe	μmol/L	♂ 14-32
		♀ 11-29
RBC	mln/μL	♂ 3.91-5.11
		♀ 3.91-5.61
HGB	g/dL	♂ 13.2-17.3
		♀ 11.7-15.5
HCT	%	♂ 38-51
		♀ 33-45
MCV	fL	86-98
MCH	pg	28-33
MCHC	g/dL	32-36

*fasting

Table S3. Reference ranges of main biochemical (lactates, fasting glucose, free Fe) and morphological (RBC, HGB, HCT, MCV, MCH, MCHC) parameters in adults, with a division based on gender (♂ - males, ♀ - females) for free Fe, RBC, HGB and HCT values. Zapala B. et al. Laboratory Diagnostics with Elements of Clinical Biochemistry. Book, 2017:874-886.

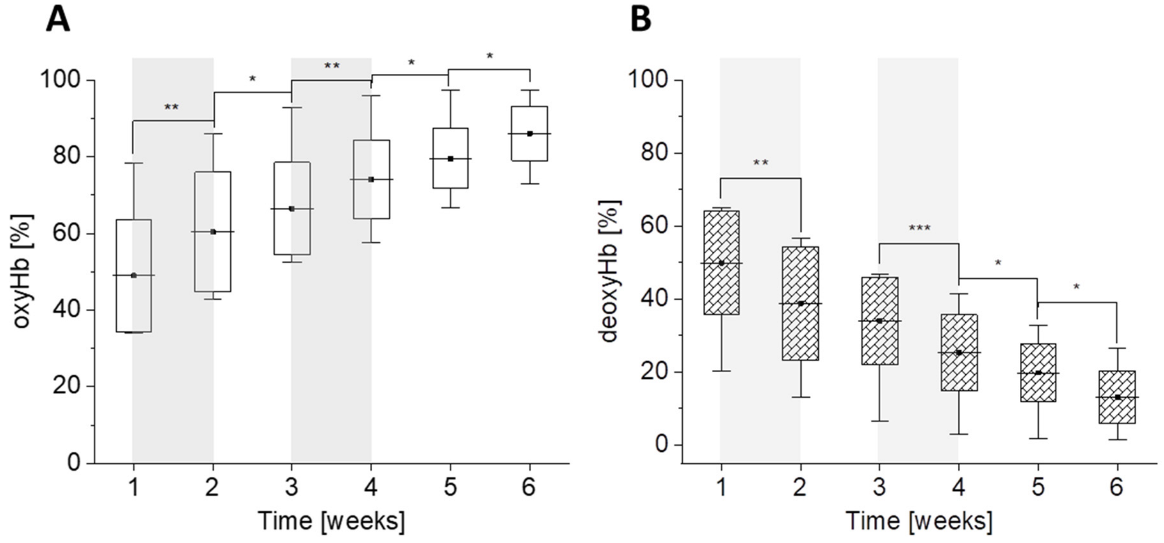

Figure S1. Percentage changes of the oxyHb (**A**) and deoxyHb (**B**) during storage pRBCs. The most prominent changes are marked gray. Data distribution is presented as box plots (mean value, mean ± SD, min-max). Statistical significance of the obtained values was tested with Kruskal-Wallis ANOVA nonparametric test (null = not significant;; *p <0.05; **p <0.01, ***p <0.001).

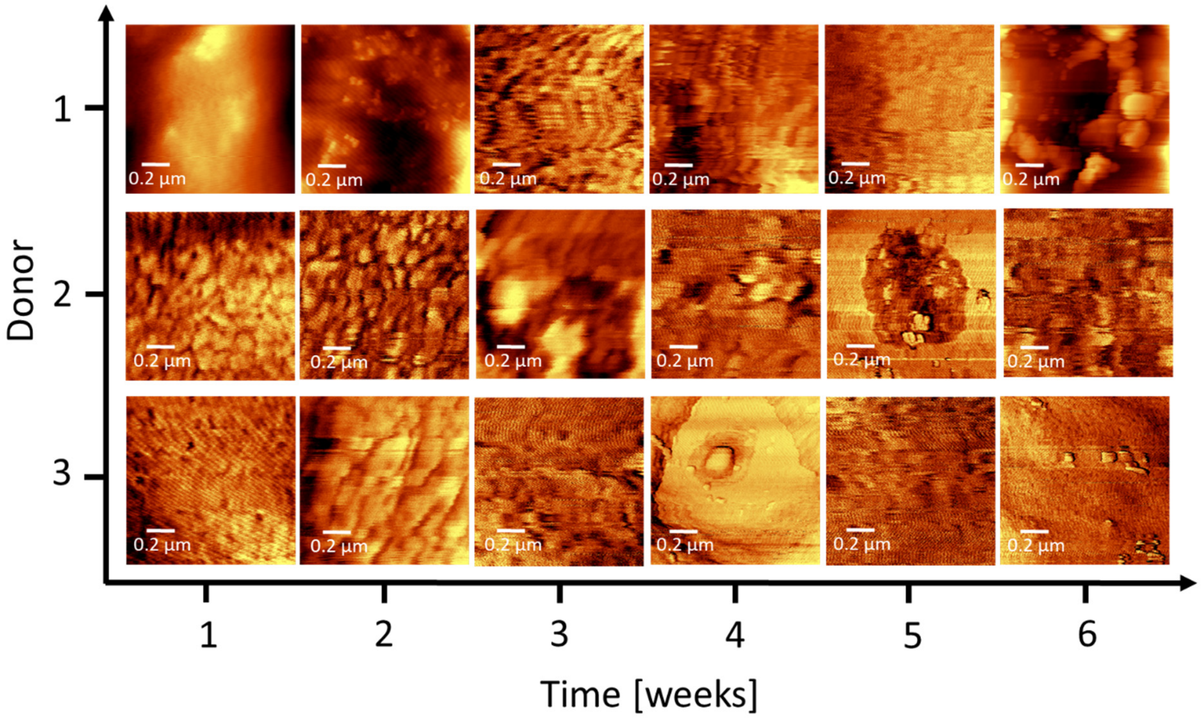

Figure S2. Representative nanoscale AFM images of RBCs' surface changes collected in scale 1.5 × 1.5 μm^2 during 6 weeks storage of the pRBCs for three donors.
